# Study of CHF_3_/CH_2_F_2_ Adsorption Separation in TIFSIX-2-Cu-i

**DOI:** 10.3390/molecules29081721

**Published:** 2024-04-11

**Authors:** Shoudong Wang, Lei Zhou, Hongyun Qin, Zixu Dong, Haoyuan Li, Bo Liu, Zhilu Wang, Lina Zhang, Qiang Fu, Xia Chen

**Affiliations:** 1 School of Chemistry and Chemical Engineering, Shandong University of Technology, Zibo 255049, China; jokemaker_wxs@163.com (S.W.); hongyunqin@163.com (H.Q.); qilin199823@sina.com (Z.D.); lhy_1999@foxmail.com (H.L.); liubo1245935971@163.com (B.L.); sevenwangzl@163.com (Z.W.); zln20000521@163.com (L.Z.); 2 Shandong Dongyue Organosilicon Materials Co., Ltd., Zibo 256401, China; zhoulei@dyyjg.com

**Keywords:** adsorption mechanism, CHF_3_/CH_2_F_2_ adsorptive separation, adsorption site, adsorption heat, TIFSIX-2-Cu-i

## Abstract

Hydrofluorocarbons (HFCs) have important applications in different industries; however, they are environmentally unfriendly due to their high global warming potential (GWP). Hence, reclamation of used hydrofluorocarbons via energy-efficient adsorption-based separation will greatly contribute to reducing their impact on the environment. In particular, the separation of azeotropic refrigerants remains challenging, such as typical mixtures of CH_2_F_2_ (HFC-23) and CHF_3_ (HFC-32), due to a lack of adsorptive mechanisms. Metal–organic frameworks (MOFs) can provide a promising solution for the separation of CHF_3_–CH_2_F_2_ mixtures. In this study, the adsorption mechanism of CHF_3_–CH_2_F_2_ mixtures in TIFSIX-2-Cu-i was revealed at the microscopic level by combining static pure-component adsorption experiments, molecular simulations, and density-functional theory (DFT) calculations. The adsorption separation selectivity of CH_2_F_2_/CHF_3_ in TIFSIX-2-Cu-i is 3.17 at 3 bar under 308 K. The existence of similar TiF_6_^2−^ binding sites for CH_2_F_2_ or CHF_3_ was revealed in TIFSIX-2-Cu-i. Interactions between the fluorine atom of the framework and the hydrogen atom of the guest molecule were found to be responsible for determining the high adsorption separation selectivity of CH_2_F_2_/CHF_3_. This exploration is important for the design of highly selective adsorbents for the separation of azeotropic refrigerants.

## 1. Introduction

HFCs are third-generation fluorinated gases (F-gases), a class of synthetic compounds used primarily in refrigeration and air conditioning (RAC) [1,2,3]. HFCs are potent greenhouse gases. So, their production and application must be phased down to meet the emission reduction target according to the Montreal Protocol [4,5]. Depending on the actual production and use of refrigerants, many of the HFCs currently in use are azeotropic or near-azeotropic refrigerant mixtures [6]. The current mainstream refrigerants include R-444A, R-447A, and R-448A, which are blends of HFCs (R-32, R-125, R-23, R-134a, etc.) with hydrofluoroolefins (HFOs). Difluoromethane (CH_2_F_2_, GWP = 675) and trifluoromethane (CHF_3_, GWP = 14,800) are the most common components of refrigerant mixture currently used in the refrigeration and air conditioning industry, with very high GWP [7,8]. Therefore, to control HFC emissions, the first thing is to separate the components from the mixtures efficiently. However, due to the low efficiency of cryogenic distillation to separate refrigerant mixtures, the amount of refrigerant gas recovered remains low. There are very similar physical properties and molecular dynamics diameters for CHF_3_/CH_2_F_2_ molecules, which makes the search for alternative technologies to energy-intensive distillation processes very challenging. In this study, CHF_3_/CH_2_F_2_ was chosen as a sample to study the separation mechanism to provide a reference for the subsequent separation of HFCs. Selective adsorption technology has become an attractive solution for gas separation considering energy efficiency and environmental protection [9,10,11,12]. Metal–organic framework (MOF) materials show great promise for gas storage and separation applications due to their significant advantages, such as flexible framework, tunable pore size and structure, and ultra-high specific surface area [13,14,15,16,17,18,19].

However, the practical applications of some MOFs are limited by their poor structural stability due to strong dependence on solvent molecules. The framework structure will collapse if they are exposed to air, high-strength acids, and bases for a period of time. TIFSIX-2-Cu-i is easy to regenerate and thermally stable under air atmosphere [20,21]. The efficient separation of C_2_H_2_/C_2_H_4_ by TIFSIX-2-M-i has been demonstrated by previous studies [20,22,23]. Inspired by these findings, TIFSIX-2-Cu-i was chosen to study the separation of CHF_3_–CH_2_F_2_ mixtures. To our knowledge, previous work on the mechanism of CHF_3_/CH_2_F_2_ adsorption in TIFSIX-2-Cu-i is sparse, which is disadvantageous to understanding and predicting interactions between adsorbates and adsorbents. In this study, the feasibility of selective separation of CHF_3_/CH_2_F_2_ by TIFSIX-2-Cu-i was evaluated for the first time. The method of adding polarization to a generic forcefield was used to obtain simulated and experimentally consistent adsorption isotherms, ensuring the accuracy of the forcefield. In this work, TIFSIX-2-Cu-i exhibits preferential adsorption of CH_2_F_2_ over CHF_3_ with a high CH_2_F_2_ adsorption capacity (2.70 mmol/g at 298 K and 1 bar). Thermodynamic and kinetic analyses were carried out by a combination of adsorption experiments and molecular simulations. The adsorption selectivity, isosteric adsorption heat, and binding sites were investigated. In addition, these works are of great significance for exploring the adsorption separation of HFCs by fluorinated anion MOFs.

## 2. Results and Discussion

### 2.1. Adsorption Isotherm

**Figure 1:**
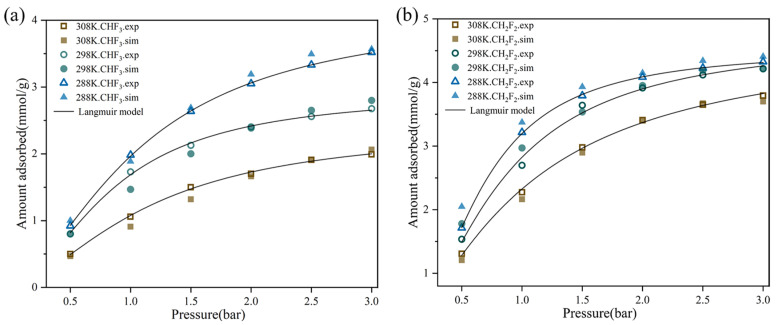
The experimental and simulated adsorption isotherms for pure CHF_3_ (**a**) or CH_2_F_2_ (**b**) at 288, 298, and 308 K in TIFSIX-2-Cu-i.

To verify the accuracy of the forcefield, the present work compares the simulation adsorption isotherms for pure CHF_3_ or CH_2_F_2_ in TIFSIX-2-Cu-i at 288, 298, and 308 K with experimental adsorption isotherms (Figure 1). The simulation results under pressure values from 0 bar to 3 bar are in good agreement with the experimental data. Therefore, it can be inferred that the potential model and polarization forcefield parameters used are reliable for predicting the adsorption of CHF_3_ and CH_2_F_2_. For all three temperatures, the trends of the CHF_3_ and CH_2_F_2_ isotherms are similar. These adsorption isotherms were fitted with the Langmuir isotherm model. The Langmuir equation is defined as shown in Equation (1). The parameters of the isotherms for CHF_3_ and CH_2_F_2_ are summarized in Table 1.

q_e_ = q_m_bp/(1 + bp) (1)

where q_e_ is the equilibrium adsorption capacity, q_m_ is the maximum adsorption capacity, b is the adsorbate–adsorbent affinity coefficient, and p is the equilibrium pressure. From Table 1, parameter b decreases with increasing temperature at the same adsorbate; parameter b is consistently larger for CH_2_F_2_ than for CHF_3_ at all three temperatures. The interaction of CH_2_F_2_ with the framework was stronger compared to CHF_3_. Parameter q_m_ shows a larger maximum adsorption capacity for CH_2_F_2_.

In detail, the uptake of CHF_3_ and CH_2_F_2_ reached 1.73 and 2.70 mmol/g at 1 bar and 298 K, respectively. At 308 K and 3 bar, the uptake of CH_2_F_2_ was 3.79 mmol/g, which is almost double the CHF_3_ uptake (1.99 mmol/g). At the same temperature and pressure, the adsorption capacity of CH_2_F_2_ was higher than that of CHF_3_. Thus, there was a greater adsorption affinity for CH_2_F_2_ than CHF_3_. The adsorption capacity of both CHF_3_ and CH_2_F_2_ increased significantly with increasing pressure. However, the adsorption capacity of CH_2_F_2_ increased faster compared to that of CHF_3_. These findings suggest that TIFSIX-2-Cu-i has stronger binding ability regarding CH_2_F_2_, indicating that TIFSIX-2-Cu-i is a potential material to separate CHF_3_/CH_2_F_2_ mixtures with high efficiency.

### 2.2. Adsorption Selectivity and Heat

In this section, thermodynamic adsorption selectivity and isosteric adsorption heat were explored, which were calculated from experimental measurements. In addition, molecular dynamics simulations were performed to explore the diffusivity of guest molecules in TIFSIX-2-Cu-i.

**Figure 2:**
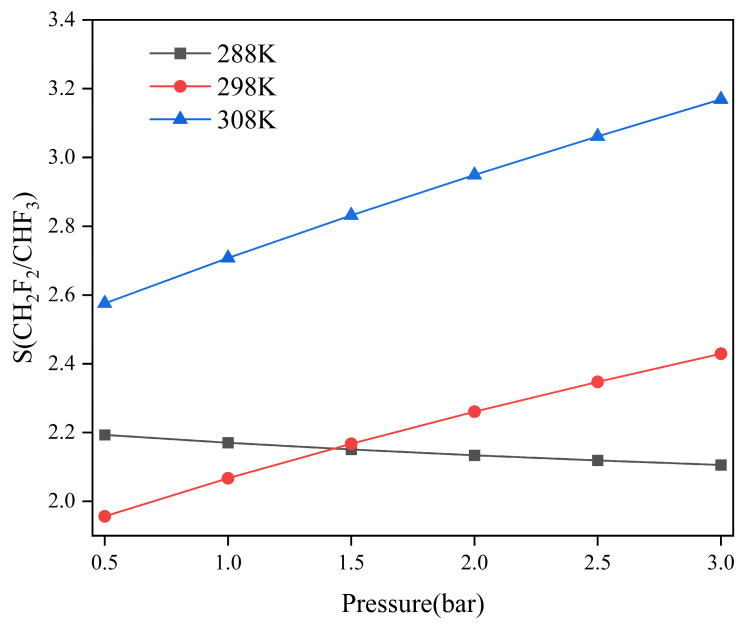
The adsorption separation selectivity for CH_2_F_2_ over CHF_3_ in TIFSIX-2-Cu-i at different temperatures and pressures, which correspond to the CH_2_F_2_-CHF_3_ mixture (CH_2_F_2_/CHF_3_, 50/50, *v*/*v*).

Myers and Praunitz developed ideal adsorbed solution theory (IAST). Here, the multicomponent adsorption equilibrium of CHF_3_ or CH_2_F_2_ was predicted using ideal adsorbed solution theory (IAST), which was calculated by the following equation [24]:

S = (x1/x2)/(y1/y2)(2)

where S is the selectivity of a component versus another one (e.g., CH_2_F_2_/CHF_3_), x is the molar fraction in the adsorbed phase, and y is the molar fraction in the gas phase. The relatively high adsorption separation selectivity is shown in Figure 2. The selectivity of CH_2_F_2_/CHF_3_ is greater than 1.0 at all the adsorption isotherms, indicating that TIFSIX-2-Cu-i preferentially adsorbs CH_2_F_2_. At 298 K and 308 K, selectivity increases with increasing pressure, while, at 288 K, selectivity decreases slightly with increasing pressure. At 288 K, the adsorption amount of CH_2_F_2_ in the high-pressure zone flattens more as pressure rises compared to CHF_3_. We hypothesize that most of the adsorption sites of the framework were then occupied by CH_2_F_2_ molecules, making it difficult for the newly added CH_2_F_2_ molecules to find available adsorption sites, leading to slowdown of the adsorption rate.

**Figure 3:**
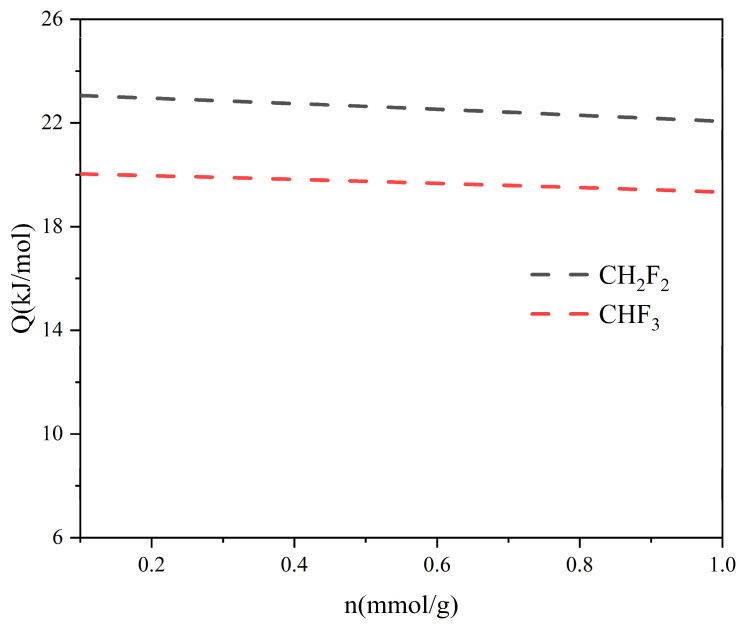
The isosteric adsorption heat (Qst) of CH_2_F_2_/CHF_3_ on the TIFSIX-2-Cu-i.

To assess the interaction strength between the framework and gas molecules, we utilized single-component isotherms obtained at three distinct temperatures (Figure 1) to determine the isosteric adsorption heat (Qst) of CH_2_F_2_/CHF_3_ on TIFSIX-2-Cu-i. The isosteric adsorption heat was calculated indirectly using the Clausius–Clapeyron equation [25]:

(3) $$\frac{d\;lnP}{dT}=\frac{q_{i}\;}{RT}$$

where *q_i_* refers to the isosteric heat of adsorption, kJ/mol; P is the pressure, MPa; T is the temperature, K; and R is the gas constant, 8.314 J/(mol·K). As shown in Figure 3, the Qst values of CHF_3_ and CH_2_F_2_ were around 20 and 23 kJ/mol^−1^. The heat of adsorption of CH_2_F_2_ was always higher than the heat of adsorption of CHF_3_ in TIFSIX-2-Cu-i. Therefore, it is directly verified that TIFSIX-2-Cu-i interacts more strongly with CH_2_F_2_ than CHF_3_, which leads to greater adsorption of CH_2_F_2_ than CHF_3_.

**Figure 4:**
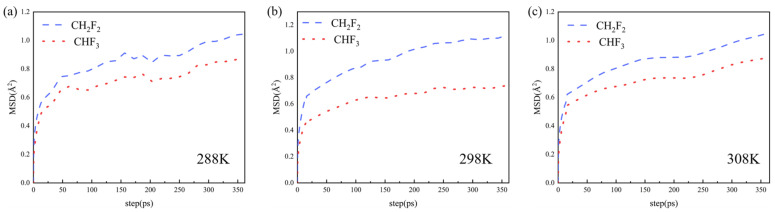
Mean-square displacements for CHF_3_–CH_2_F_2_ mixture (CHF_3_/CH_2_F_2_, 50/50, *v*/*v*) in TIFSIX-2-Cu-i at 288 K (**a**), 298 K (**b**), and 308 K (**c**).

In this part, the free diffusion behavior of CHF_3_ and CH_2_F_2_ in TIFSIX-2-Cu-i was explored. The corresponding mean square displacements (MSDs) obtained from the simulations are shown in Figure 4. The self-diffusion coefficients of CHF_3_ and CH_2_F_2_ in the TIFSIX-2-Cu-i were calculated using the Einstein relation as shown below:

(4) $$D=\frac{1}{6}\underset{n\to \infty }{\lim}\frac{d}{dt}\left\{\frac{1}{N_{i}}\sum_{i=1}^{N_{i}}\langle {\left|r_{i}\left(t\right)-r_{i}\left(0\right)\right|}^{2}\rangle \right\}$$

where the average is taken over time t for the mean square displacement of the center of mass position vectors r of all the molecules N in the system; ‹›indicates the overall average. The calculated results show that the self-diffusion coefficients of CHF_3_ and CH_2_F_2_ are 1.15 × 10^−4^ cm^2^/s and 2.18 × 10^−4^ cm^2^/s, respectively. This finding showed that TIFSIX-2-Cu-i exhibited high kinetic selectivity for CH_2_F_2_ over CHF_3_. TIFSIX-2-Cu-i is a doubly interpenetrated framework attributed to the much longer organic linker 4,4-dipyridylacetylene and slightly large pore sizes of about 5.2 × 5.2 Å. That makes it easier for the mixture to diffuse into the adsorption sites within the pores. The properties of this MOF can be characterized as pillared square lattice networks with a pcu topology, attributed to their pore surfaces with narrow pore sizes and highly electrostatic pore surfaces. These features combined provide exceptionally strong binding interactions with polarizable molecules, such as CHF_3_ and CH_2_F_2_ [26]. This also enabled the two guest molecules to possess high adsorption capacity.

### 2.3. Adsorption Sites

**Figure 5:**
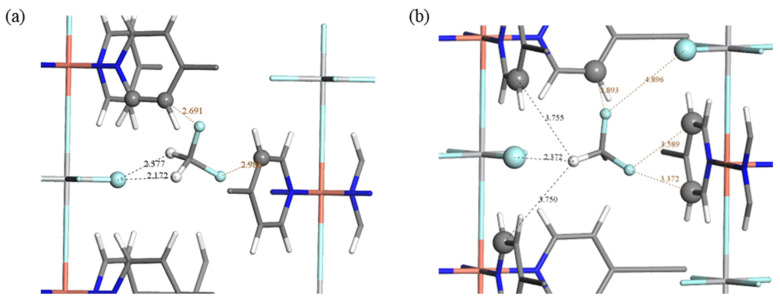
The typical binding sites for CH_2_F_2_ (**a**) or CHF_3_ (**b**) in TIFSIX-2-Cu-i. The cyan, gray, and white spheres represent fluorine, carbon, and hydrogen atoms, respectively.

Figure 5 shows the optimal adsorption binding sites for CHF_3_ and CH_2_F_2_. The snapshots obtained detailed information about the adsorption of pure CHF_3_ and CH_2_F_2_ in TIFSIX-2-Cu-i at 298 K and 1 bar. In the doubly interpenetrated framework of TIFSIX-2-Cu-i, the H atom of CHF_3_ binds with the F atom from TiF_6_^2−^. The distance of the C–H⋯F hydrogen bond was 2.372 Å (Figure 5b). The H⋯F distance of 2.372 Å obtained by the simulation was smaller than the sum of the van der Waals radii of H and F (2.55 Å) [27], confirming the existence of electrostatic interactions for H^δ+^⋯F^δ−^. This was consistent with the reported binding sites of TIFSIX-2-Cu-i to short-chain alkanes (C_2_H_2_, C_2_H_4_) [20,22,23]. CH_2_F_2_ has a similar binding site. The two H atoms of CH_2_F_2_ are bound at the F site by virtue of a synergistic hydrogen bonding interaction. The shortest length of the C–H⋯F bond between CH_2_F_2_ and the TIF_6_^2−^ site is 2.172 Å, which is shorter than the C–H⋯F between CHF_3_ and TIF_6_^2−^. The TIFSIX-2-Cu-i interacts more strongly with CH_2_F_2_ than CHF_3_.

**Figure 6:**
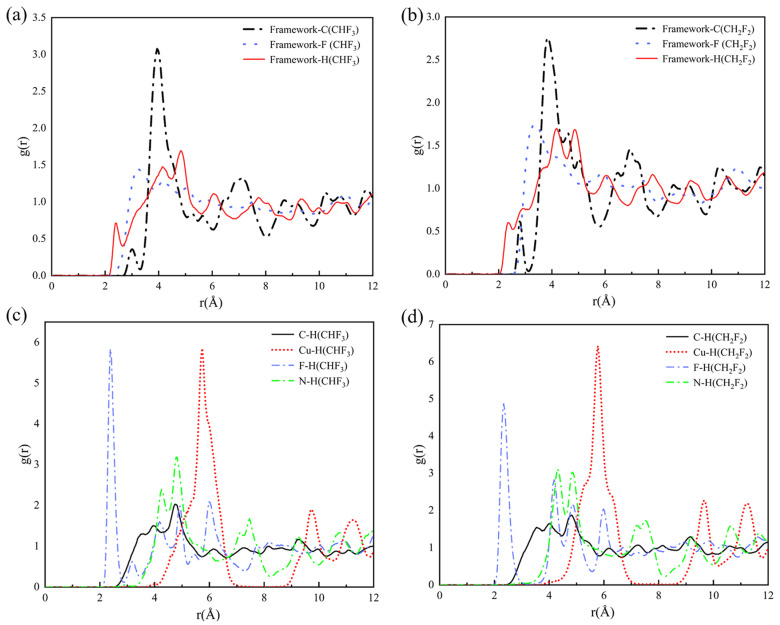
The RDF between the framework and each atom of CHF_3_ (**a**)/CH_2_F_2_ (**b**); the RDF between the representative atoms on the framework and hydrogen atom of CHF_3_ (**c**)/CH_2_F_2_ (**d**) in 298 K.

Radial Distribution Functions (RDFs) amount to one of the most common methods for determination of interatomic distances. The RDFs in 298 K describing the interactions between the individual atoms of the pure CHF_3_/CH_2_F_2_ and the TIFSIX-2-Cu-i framework are shown in Figure 6a,b. In Figure 6a, the framework interacts preferentially with H in CHF_3_. From the investigation of the RDF of H (CHF_3_) with each atom of the framework in Figure 6c, it was found that F (framework) interacts preferentially with H (CHF_3_), which is the same as the snapshot conclusion of Figure 5a. Similarly, according to Figure 6b,d, it is found that F (framework) interacts preferentially with H (CH_2_F_2_). Compared to CHF_3_, CH_2_F_2_ has more H–F bonds interacting with the framework at the same time. So, we believe that the reason for the higher adsorption affinity of CH_2_F_2_ than CHF_3_ is due to the binding geometry of CH_2_F_2_/CHF_3_ adsorbed in the supercage of TIFSIX-2-Cu-i.

### 2.4. Redistribution of Charge Density

**Figure 7:**
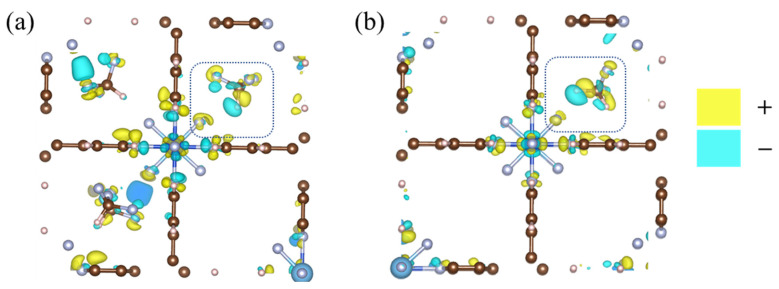
The redistribution of charge density in TIFSIX-2-Cu-i after adsorbing CHF_3_ molecules (**a**) or CH_2_F_2_ molecules (**b**). Color code: brown, C; meat pink, H; silver, F; blue, Ti.

**Figure 8:**
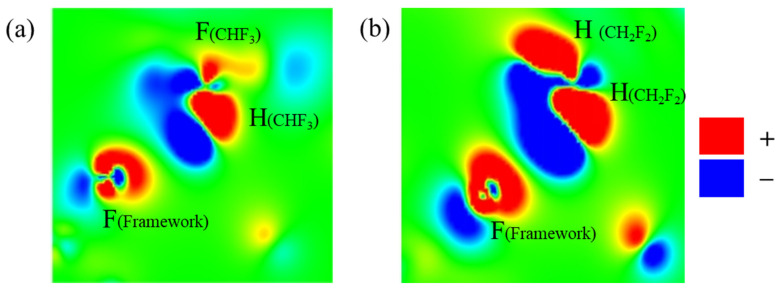
The slices of charge density redistribution for TIFSIX-2-Cu-i and CHF_3_ (**a**)/CH_2_F_2_ (**b**) after molecular adsorption, which correspond to the electron transfer between hydrogen atom of CHF_3_/CH_2_F_2_ and the fluorine atom of TIF_6_^2−^.

In order to study the charge change in TIFSIX-2-Cu-i after adsorption, DFT calculations were carried out to investigate the redistribution of charge density in this system after adsorption of CHF_3_/CH_2_F_2_ molecules. As shown in Figure 7, the electrons of the H atoms of CHF_3_/CH_2_F_2_ migrate to the F atoms of the framework due to the strong electron-withdrawing ability of the F atoms, where “–” denotes a negative charge. As shown in Figure 8, the blue area in Figure 8b is larger and darker than in Figure 8a, and the charge density transfer between CH_2_F_2_ and the framework F atom is more pronounced. This may be because, compared to CHF_3_, CH_2_F_2_ has more interactions between H atoms and framework F atoms, which is also the reason for the relatively high adsorption separation selectivity for CH_2_F_2_ over CHF_3_.

## 3. Experiment Section

### 3.1. Preparation of Materials

All reagents and solvents were obtained commercially and used as received without further purification. Copper fluoroborate [Cu(BF_4_)_2_, 98.5%], methanol (CH_3_OH, 99%), and ammonium hexafluoro titanate ((NH_4_)_2_TiF_6_, 98%) were bought from Aladdin; 1,2-Di(pyridin-4yl)ethyne (C_12_H_8_N_2_, 99.35%) was purchased from Leyan Co., Ltd. (Shanghai, China); and helium (He, 99.999%) gas was purchased from BaiYan Co., Ltd. (Zibo, China); The methylene fluoride (CH_2_F_2_, 99.999%) and the methyl trifluoride (CHF_3_, 99.999%) were obtained from Dong Yue Co., Ltd. (Zibo, China).

### 3.2. Synthetic Procedures

Cu(BF_4_)_2_ (1 mmol), (NH_4_)_2_TiF_6_ (1 mmol), and 1,2-Di(pyridin-4yl) ethyne (2 mmol) were dissolved in 5 mL of water and 10 mL of methanol, and a blue slurry product was obtained after stirring at 338 K for 12 h. Then, the slurry was filtered and washed with 10 mL of methanol. The blue filter cake was heated at 393 K for 12 h under vacuum conditions to obtain TIFSIX-2-Cu-i material [26].

### 3.3. Characterization

**Figure 9:**
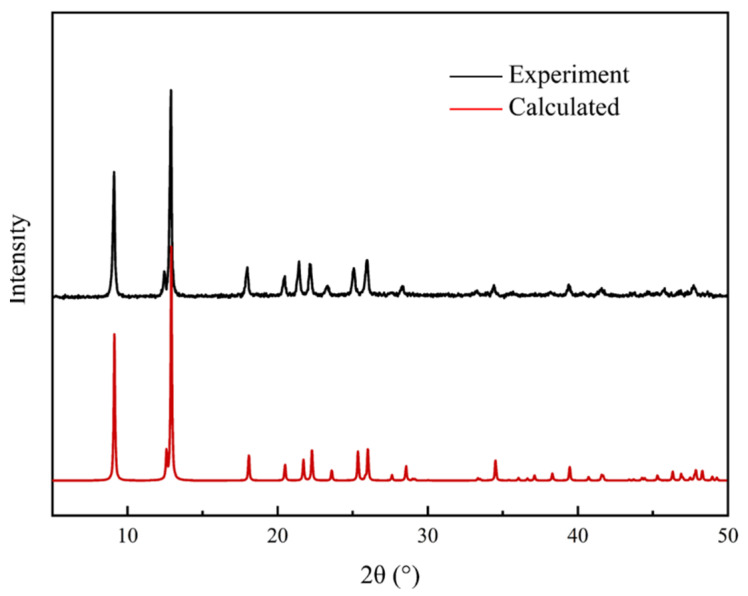
XRD patterns of TIFSIX-2-Cu-i after activation (compared to calculated patterns).

The activated TIFSIX-2-Cu-i was subjected to X-ray diffraction characterization and scanned using a Bruker AXS D8 ADVANCE diffractometer under a CuKα radiation source operated at a voltage of 40 kV, a current of 20 mA, and a scattering angle in the range (2θ) of 5–40 degrees. The XRD pattern of TIFSIX-2-Cu-i was presented in Figure 9, which was compared to calculated patterns. ASAP 2460 (Micromeritics company, Shanghai, China) was employed in this experiment. Pore structure was characterized by the N_2_ adsorption method. The experimental temperature was 77 K. Before testing, the sample was treated by 12 h vacuumization at 393 K. Specific surface area was calculated using a multipoint Brunauer–Emmett–Teller model (BET). Table 2 shows the pore parameters of TIFSIX-2-Cu-i.

### 3.4. Single-Component Adsorption Measurements

**Figure 10:**
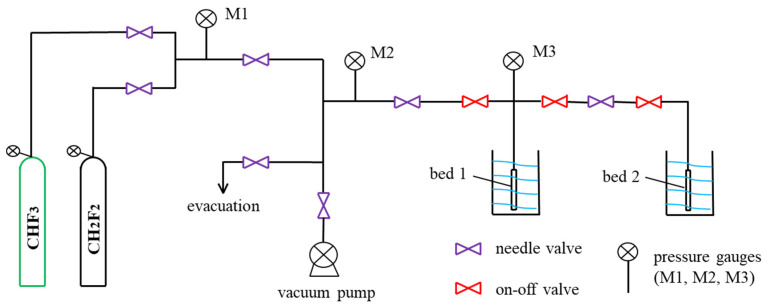
Diagram of adsorption measurements’ experimental apparatus.

The adsorption isotherms of CHF_3_ and CH_2_F_2_ were measured in the absolute pressure range of 1–3 bar in TIFSIX-2-Cu-i framework. The experimental temperatures were 288, 298, and 308 K. Excess adsorption experiments were performed using activated TIFSIX-2-Cu-i monomer. The temperature was controlled by an external circulating water bath. Before the measurements, TIFSIX-2-Cu-i was degassed at 393 K for 12 h under vacuum conditions. CHF_3_ and CH_2_F_2_ gas of purity 99.99% were used as adsorbates. The adsorption capacity of pure CHF_3_ or CH_2_F_2_ was calculated based on the pressure changes before and after adsorption. Figure 10 shows the diagram of adsorption measurements’ experimental apparatus. A standard volumetric method was used to measure pure gas adsorption isotherms [28]. The homemade apparatus is designed by the proposed method.

## 4. Models and Methods

### 4.1. Models

**Figure 11:**
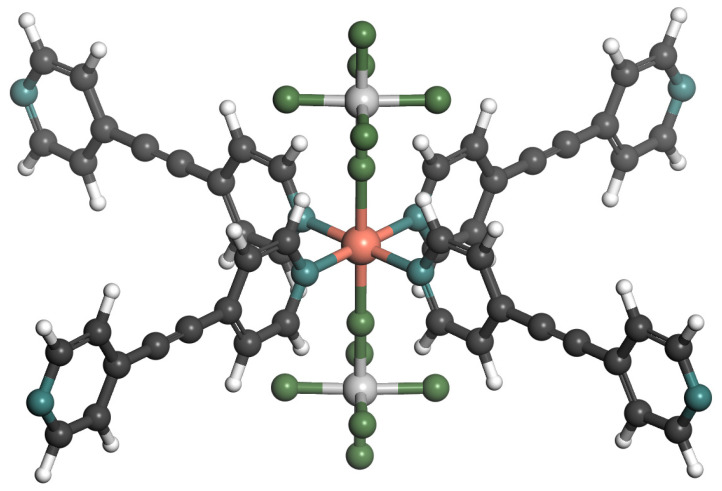
The chemically different atoms in TIFSIX-2-Cu-i. Atom colors: C = gray; H = white; N = blue; F = green; Ti = silver; Cu = red.

The interpenetrated polymorph, TIFSIX-2-Cu-i, is composed of doubly interpenetrated nets that are isostructural to the nets in TIFSIX-2-Cu. The independent nets are staggered with respect to one another, affording 5.2 Å pores [22,23]. The crystal cells used in the simulation were downloaded from the Cambridge Crystallographic Data Center (CCDC) as structural files. Optimized structure by DFT simulation was used for further calculations. TIFSIX-2-Cu-i is a variant of SIFSIX-2-Cu-i. Ti^4+^ has a higher polarizability relative to Si^4+^ [29]. So, TIFSIX-2-Cu-i has a higher thermal stability (decomposition temperature of 262 °C), which may be attributed to the relatively higher polarizability of Ti^4+^ [20]. TIFSIX-2-Cu-i atoms in the framework with different chemical properties are shown in Figure 11.

### 4.2. Density Functional Theory Calculations

The MOFs’ structure was optimized using ab initio density functional theory (DFT) as implemented in the Vienna Ab Initio Simulation Package version 2.2 (VASP) [30], with the overall energy converged to within 10^−5^ eV per atom. The Perdew–Burke–Ernzerhof (PBE) function of the generalized gradient approximation (GGA) [31] was used to represent the electron exchange correlation, and a cutoff energy of 500 eV was set for the plane wave. According to the Monkhorst–Pack methodology, the Brillouin zone was sampled with a series of K-point grids (2 × 2 × 4). After geometrical optimization, we obtained the electron charge density and then used Density Derived Electrostatic and Chemical (DDEC6) [32,33] to calculate the atomic charge of the net atomic charge framework for each MOF atom (Table 3). DFT simulations were used to explore the redistribution of charge density in this system after adsorption of CHF_3_ or CH_2_F_2_ molecules using the CP2K code [34]. The DZVP-MOLOPT-SR-GTH [35] basis set and the Goedecker–Teter–Hutter [36] pseudopotential were used, and the density generalization employed was a PBE with DFT-D3 [37] dispersion corrections.

### 4.3. Grand Canonical Monte Carlo Simulations

All GCMC simulations were performed with the RASPA [38] code to study CHF_3_/CH_2_F_2_ adsorption properties under different conditions. A grand canonical systematic (μVT) was used, where the system was under constant chemical potential, volume, and temperature. To eliminate periodic boundary conditions, we used supercell by 2 × 2 × 3 replicas of the unit cell for the calculations. The van der Waals interactions were truncated to a radius of 12 Å, and tail corrections were used to approximate the contributions beyond this truncation. In the simulations, 1 × 10^6^ Monte Carlo steps were used for the equilibration and 1 × 10^7^ Monte Carlo steps were used for production runs. The adsorbate molecules and the adsorbate framework were treated as rigid structures. CHF_3_ and CH_2_F_2_ were modeled as rigid tetrahedral molecules with five charged interaction sites. From previous simulation study, rigid body models were used to represent molecules as a collection of fixed geometric shapes that maintain a constant structure and orientation throughout the simulation. The host–guest and guest–guest interactions in the system were described by the short-range force and the electrostatic force, which are described by the Lennard-Jones and Coulomb potential functions (Equation (5)):

(5) $$U_{inter}\left(r_{ij}\right)=4\varepsilon_{ij}\left[{\left(\frac{\sigma_{ij}}{r_{ij}}\right)}^{12}-{\left(\frac{\sigma_{ij}}{r_{ij}}\right)}^{6}\right]+\frac{1}{{4\pi \varepsilon }_{0}}\frac{q_{i}q_{j}}{r_{ij}}$$

where $j\sigma_{ij}$ and $\varepsilon_{ij}$ are the collision diameter and potential well depth, respectively, $r_{ij}$ is the distance between sites i and j, $q_{i}$ denotes the atomic charge on site i, and $\varepsilon_{0}$ is the permittivity of free space. The cross-interactions with other molecules and frameworks are obtained using the Lorentz–Berthelot mixing rule (as shown in Equations (6) and (7)):

(6) $$\sigma_{ij}=\frac{\sigma_{ii}+\sigma_{jj}}{2}$$

(7) $$\varepsilon_{ij}=\sqrt{\varepsilon_{ii}\cdot \varepsilon_{jj}}$$

A polarizable forcefield was employed to achieve an accurate description of the adsorption behavior of CHF_3_/CH_2_F_2_ in TIFSIX-2-Cu-i for molecular simulations. Back-polarization was neglected to achieve reasonable simulation times. To account for the implied polarization, we rescaled the Lennard-Jones [39] energy parameters according to the atomic polarizabilities. The Lennard-Jones energy parameters and charges of CHF_3_ and CH_2_F_2_ were taken from previous studies [40,41]. The Lennard-Jones parameters of TIFSIX-2-Cu-i for N, C, and H were taken from the OPLS-AA forcefield, and the rest of the atoms were taken from the UFF–Dreiding hybrid forcefield [42]. The equations used to adjust the parameters in this study are as follows:

(8) $$\varepsilon_{i}^{scaled}=\varepsilon_{i}\cdot \frac{\left(1+\lambda \right)-\frac{\alpha_{i}}{\alpha_{max}}}{\left(1+\lambda \right)-\frac{\alpha_{i}}{\alpha_{max}}\cdot \lambda }$$

*α_i_* means the polarizability of atom_i_, *α_max_* means the max polarizability, and *ε_i_* means the initial forcefield parameter. *λ* and *ξ* are scaling factors between 0 and 1, whose values depend on the discrepancy between the experimental data and the simulation results, used to rescale the Lennard-Jones energy parameters. The detailed methodology is described in Refs. [43,44]. In this study, by fitting the experimental data to the simulation results, *λ* was set to 0.2 and *ξ* to 0.9 for CH_2_F_2_, while λ was set to 0.9 and *ξ* to 0.01 for CHF_3_. Table 3 and Table 4 summarize all the forcefield parameters, atomic polarizabilities [45,46], and atomic charges.

### 4.4. Molecular Dynamics Simulations Details

In this paper, RASPA code was used to perform molecular dynamics simulations of CHF_3_/CH_2_F_2_ in TIFSIX-2-Cu-i. The polarized forcefield parameters from Table 3 and Table 4 were employed. All MD simulations were employed for 1 ns with a time step of 0.5 fs in the NVT ensemble to explore the diffusion of the equimolar CHF_3_/CH_2_F_2_ mixture in TIFSIX-2-Cu-i. The simulations were performed for 1 × 10^7^ cycles, 2000 initialization cycles, and 20,000 equilibration cycles. We truncated the van der Waals interaction with a radius of 12.0 Å and used tail correction to approximate the contribution beyond this cutoff.

## 5. Conclusions

In this study, the adsorption mechanisms of CHF_3_ or CH_2_F_2_ in the TIFSIX-2-Cu-i framework were studied by combining single-component adsorption experiments and molecular simulations. In order to ensure consistency between the experimental data and the simulation results, a polarization forcefield was introduced. TIFSIX-2-Cu-i has excellent CH_2_F_2_ adsorption capacity (3.79 mmol/g) and CH_2_F_2_/CHF_3_ selectivity (3.17) at 3 bar and 308 K, making it a promising material to separate CHF_3_/CH_2_F_2_ mixtures. Regarding the competitive adsorption of CHF_3_–CH_2_F_2_ mixtures in TIFSIX-2-Cu-i, both the thermodynamic and kinetic selectivity of CH_2_F_2_ relative to CHF_3_ were observed to be relatively high. According to the combined effect of adsorption and diffusion, TIFSIX-2-Cu-i exhibits markedly preferential adsorption of CH_2_F_2_ for CHF_3_. The calculated heats of adsorption indicate relatively strong interactions between CH_2_F_2_ and TIFSIX-2-Cu-i. The snapshots show that CHF_3_/CH_2_F_2_ adsorption on TIFSIX-2-Cu-i involves multiple H⋯F interactions, where CHF_3_/CH_2_F_2_ interacts with TiF_6_^2−^ simultaneously. The typical binding sites of CH_2_F_2_ molecules in the TIFSIX-2-Cu-i channel are very similar to those of CHF_3_, and the F atoms in the TiF_6_^2−^ of the TIFSIX-2-Cu-i framework preferentially adsorb with H atoms of the CH_2_F_2_ molecule. CH_2_F_2_ has more H atoms, and TIFSIX-2-Cu-i shows stronger affinity for CH_2_F_2_ than CHF_3_.

In conclusion, this study reveals the adsorption mechanism of CHF_3_–CH_2_F_2_ mixtures in TIFSIX-2-Cu-i channels at the microscopic level. This research exploration provides an effective and superior strategy for the design and screening of highly selective adsorbents for the separation of CHF_3_–CH_2_F_2_ mixtures.

## Figures and Tables

**Table 1:** Parameters of CHF_3_ and CH_2_F_2_ fitted by the Langmuir Adsorption Isotherm Model.

	T (K)	q_m_ (mmol/g)	b (bar^−1^)	R-Squared
	288	6.22417	0.85526	0.9582
CH_2_F_2_	298	7.06367	0.57236	0.97048
	308	6.97756	0.44233	0.9712
	288	4.92555	0.51897	0.976526
CHF_3_	298	4.22734	0.47127	0.96737
	308	3.8638	0.38144	0.9911

**Table 2:** Pore parameters of TIFSIX-2-Cu-i.

S_BET_ (m^2^/g)	Sample Density (cm^3^/g)	Total Pore Volume (cm^3^/g)	t-Plot Micropore Volume (cm^3^/g)	Mesopore Volume (cm^3^/g)	Average Pore Size (Å)
372	1.211	0.211	0.131	0.079	3.988

**Table 3:** Partial charges, forcefield parameters, and atomic polarizabilities corresponding to the atom types in CHF_3_ and CH_2_F_2_.

Atom Types	*q* (|e|)	*σ* (Å)	*ε* (K)	*α* (Å^3^)
C_CHF_3_	0.719	3.52	55	1.2886
H_CHF_3_	0.016	2.6	25.2	0.4138
F_CHF_3_	−0.245	2.92	25	0.44475
C_CH_2_F_2_	0.385	3.46	42	1.2886
H_CH_2_F_2_	0.049	2.2	29	0.4138
F_CH_2_F_2_	−0.2415	2.95	37	0.44475

**Table 4:** Forcefield parameters and the atomic polarizabilities corresponding to the atom types in the TIFSIX-2-Cu-i.

Atom Types	σ (Å)	ε (K)	α (Å^3^)
N	3.25	85.5479	0.97157
C	3.5	40.258	1.2886
H	2.42	15.097	0.4138
F	3.0932	36.4834	0.44475
Cu	3.114	2.51	2.1963
Ti	2.8286	8.55473	3.2428

## Data Availability

The data presented in this study are available upon request from the corresponding author.
